# Lichens in Pharmacological Action: What Happened in the Last Decade?

**DOI:** 10.5152/eurasianjmed.2022.22335

**Published:** 2022-12-01

**Authors:** Yalçın Karagöz, Berna Öztürk Karagöz

**Affiliations:** 1 Department of Pharmaceutical Botany, Ağrı İbrahim Çeçen University Faculty of Pharmacy, Ağrı, Turkey; 2 Department of Pharmacology, Ağrı İbrahim Çeçen University Faculty of Pharmacy, Ağrı, Turkey

**Keywords:** Lichens, natural products, biologic pharmaceuticals, literature review

## Abstract

Lichens are a unique group of organisms, which can produce compounds that are named secondary metabolites and rarely or are not produced in other organisms. Lichens possess pharmacological actions related to their secondary metabolites. Our knowledge of lichens and their pharmacological actions rapidly increases as new technologies and devices, which facilitate the investigation of the chemical profile and biological activities of lichens, are introduced and become more readily available. In addition, new methods and perspectives, as well as suggestions for pharmacological mechanisms, accumulate daily. Furthermore, lichen substances stand as a relatively untapped source of natural products. Accordingly, researchers investigate the pharmacological actions of lichen-derived material more frequently than it was in the past. This review focused on the pharmacological activities of lichens published in the last 11 years (2012-2022). Literature data obtained from WebOfScience and PubMed databases using related search keywords revealed that anti-genotoxicity, anti-cancer, and anti-microbial activity studies have constantly been conducted. More recently, immunomodulatory and inflammation-related studies took to the stage. Enzyme inhibition actions were popular as well. Our selection was based on the novelty and mechanistic insight that papers presented.

## Introduction

Lichens are typically described as symbiotic organizations composed of a fungus and a photosynthetic symbiont, generally a green alga or cyanobacteria, or both. However, recent discoveries of microorganisms (bacteria or fungi) constantly present in or over lichen thalli led scientists to coin the more accurate term “miniature ecosystem.” In this “ecosystem,” chemical interactions occur between any partners, enriching the pool of lichen-derived substances. This richness eased the way of lichens into ethnobotanical/ethnopharmacological use throughout history.^1^

Lichen secondary metabolites are mainly depsides and depsidones, but also monocyclic aromatic compounds, dibenzofurans, depsones, amino-acid derivatives, aliphatic acids, macrocyclic lactones, sugar alcohols, quinones, xanthones, chromones carotenoids, and terpenoids were discovered in lichens.^2^

Pharmacological actions of lichens are unambiguously related to their secondary metabolite content. The quantity and quality of lichen substances present in any thalli were not easy to determine until recently. The advancements in analytical techniques and the rapid spread of modern analytical devices stimulated phytochemical analysis of lichens all over the world, increasing the number of purified compounds and bioactivity tests related to these compounds. In addition, the accumulated chemical data regarding lichen substances became readily available to anyone who asks for it. Considered together, these alterations in status led to folds of increase in lichen substances’ bioactivity-related papers.

This abundance of papers on pharmaceutical/pharmacological activities of lichens necessitates reviewing the literature and revisiting some of the less pronounced data. Therefore, we put together experimental papers published between 2012 and 2022, with particular interest in more recent papers. A summary of pharmacological actions of lichens is presented in Table 1. Following is the account of our selection of papers in a pharmacological activity-oriented layout.

## Pharmacological Activities of Lichens

### Anti-Mutagenic and Antioxidant Activity

A methanolic *Cladonia foliacea *extract was evaluated for antigenotoxic and antioxidant activities in various test systems. Investigators reported that the extract was not mutagenic and had antigenotoxic effect as well as a poor antioxidant capacity.^3^

Total extracts from *Letharia vulpina* and *Vulpicida pinastri *were evaluated for antigenotoxic and antioxidant capacity in human lymphocytes treated with aflatoxin B1. Authors reported that lichen extracts decreased malondialdehyde levels and micronuclei frequencies and increased glutathione peroxidase, glutathione, and superoxide dismutase levels.^4^

Methanolic extracts of *Peltigera horizontalis* and *Peltigera praetextata *were assessed for mutagenic, anti-mutagenic, and antioxidant activity. The results indicated that lichen extracts had no mutagenic activity. On the contrary, they had anti-mutagenic and antioxidant activity in all tested doses.^5^

*Cetraria aculeata, Cetrelia olivetorum,* and *Cladonia chlorophaea* extracts were shown to have significant anti-mutagenic effects in N-methyl-N′-nitro-N-nitrosoguanidine, aflatoxin B1, sodium azide, and acridin induced mutagenesis tests. The extracts also improved superoxide dismutase and glutathione peroxidase activities and decreased malondialdehyde and glutathione amounts.^6^

Using single-cell gel electrophoresis, researchers assessed antigenotoxic effects of lobaric, diffractaic, vulpinic, and lecanoric acids, on human lymphocytes in vitro. Authors reported that all lichen acids mediated a considerable decrease in total DNA damage at all tested concentrations, and vulpinic acid was best among them.^7^

Antioxidant and cytotoxic potential of *Pseudevernia furfuracea*-derived olivetoric and physodic acids were examined on cultured human amnion fibroblasts. Researchers revealed that these metabolites were not genotoxic, while they exhibited strong antioxidant potential at low doses.^8^

Researchers found that usnic acid (UA) had no mutagenic effects on human lymphocytes, and low concentrations (1 and 5 µg/mL) of UA increased total antioxidant capacity in cultured human blood cells but had no effect on total oxidative status.^9^

### Antimicrobial Activity

In an interesting study, Lauinger et al^10^ chemically targeted liver-stage parasites of *Plasmodium berghei*, the malaria parasite, with (+)-usnic, vulpic, evernic, and psoromic acids (PA). They found that UA exhibited the highest liver stage activity and stage specificity (liver stage IC50: 2.3 μM, blood stage IC50: 47.3 μM). The authors reported that other 3 compounds inhibited at least 1 enzyme (PfFabZ, PfFabG, PfFabI) from the plasmodial fatty acid biosynthesis (FAS-II) route, a possible target route. In addition, the compounds were also assessed against whole cells and FabI homologs of *Staphylococcus aureus, Mycobacterium tuberculosis, *and* Escherichia coli*. According to in silico studies, compounds acted indirectly by attaching to allosteric sites on the protein surface of the FAS-II enzymes.

Three UA derivatives and their metal salts were synthesized and tested against 10 pathogenic microorganisms. In addition, antimutagenic activity of these complexes was evaluated. Researchers reported that UA derivatives had potent antimutagenic effects and metal complexes had potent antimicrobial effects.^11^

Therapeutic action of UA against coccidiosis was tested in broilers and was compared to that of toltrazuril. Authors reported that 100 mg/kg dose of UA exhibited anticoccidial activity compared to toltrazuril.^12^

In an extensive study, Shrestha et al^13^ investigated antibiotic effects of 34 North American lichen extracts against gram (+) *S. aureus*, *Pseudomonas aeruginosa*, and methicillin-resistant *S. aureus* and gram (−) *E. coli. *Acetone extracts of *Vulpicida canadensis,* *Letharia vulpina*, and* L. columbiana *demonstrated antibacterial activity against *E. coli. *Except for *Lobaria pulmonaria,* all lichen extracts exhibited inhibitory effects against gram (+) bacteria with a range 3.9-500 µg/mL of MIC values. The effects of *Rhizoplaca marginalis* and *Usnea hirta* against methicillin-resistant *S. aureus* (7.8 µg/mL MIC) are noteworthy.

Basile et al^14^ investigated acetone extract of *Xanthoria parietina*’s antifungal, antibacterial, and antiproliferative activities and its major secondary metabolite, parietin. They found that both exhibited antibacterial potential against 9 pathogenic bacteria including standard ATCC (American Type Culture Collection) and multi-drug-resistant strains. Parietin performed better in antifungal test than acetone extract. The acetone extract also inhibited proliferation of human breast cancer cells and triggered apoptosis. This inhibition came with modification of production of cell cycle adjusting genes p16, p27, cyclin A, and cyclin D1. The extract triggered apoptosis through activation of external and internal cell death pathways, modifying various molecular processes.

Protocetraric acid isolated from *Usnea albopunctata* was found to have a supreme antimicrobial effect against a range of bacteria and fungi. It produced inhibition zones comparable to ciprofloxacin in disc diffusion tests and MIC (Minimum Inhibitory Concentration) values were better than those of ciprofloxacin for some of the tested bacteria. Especially, 0.5 µg/mL MIC value for *Salmonella typhi *is impressive. In case of fungi, protocetraric acid performed comparably to amphotericin B in disc diffusion test, but MIC values were much higher than those of amphotericin B, except for *Trichophyton rubrum, *where protocetraric acid had a MIC value of 1 µg/mL and amphotericin B had a MIC value of 4 µg/mL.^15^

Pathak et al^16^ tested acetone extract of *Usnea orientalis *against 6 dermatophyte species and found that the extract produced MIC values between 9 and 1040 µg/mL. Authors revealed the presence of usnic and salazinic acids in the extract.

According to a recent study,^17^ *Usnea steineri *acetone extract displayed strong antibacterial effect (<10 µg/mL) against resistant *Staphylococcus epidermidis* and *Enterococcus faecalis* strains. Authors isolated (+) UA, which displayed potent activity against *S. aureus* and *S. haemolyticus* (MIC: 12.5 µg/mL) and particularly *S. epidermidis *(MIC: 3.12 µg/mL). Another interesting finding was the absence of synergistic antimicrobial effects in combinations of (+)-UA with penicillin and tetracycline.

The authors studied 27 secondary lichen metabolites isolated from 28 lichen species for their ability to inhibit RecA, an essential protein for genetic recombination, from *E. coli*. They found that 9 compounds exhibited over 80% inhibition of ATP hydrolytic activity of RecA protein. Among those 9 (IC50 values ranging from 14.2 to 42.6 µM) compounds, authors put forward epiphorellic acid, isolated from *Cornicularia epiphorella,* as a propitious candidate for the development of more potent RecA inhibitors.^18^

Karagoz et al^19^ tested extract fractions of *Bryoria capillaris* against numerous human and plant pathogens. They found that fractions rich in barbatolic and alectorialic acids were effective antimicrobial agents against tested bacteria in liquid but not in solid media.

In a recent study^20^, divaricatic acid (DA) was isolated from *Evernia mesomorpha *and discovered to be effective against gram (+) bacteria with MIC values comparable to vancomycin. Interestingly, DA performed better than vancomycin against *Staphylococcus epidermidis* and *Enterococcus faecium* and was active against *Candida albicans*. Moreover, DA was as active as vancomycin against methicillin-resistant *S. aureus*.

Hassan et al^21^ investigated anti-mycobacterial activity of PA, a common lichen secondary metabolite, against clinical *M. tuberculosis* strains. Additionally, the inhibitory action of PA on UDP-galactopyranose mutase (UGM) and arylamine-N-acetyltransferase (TBNAT), 2 critical enzymes associated with *M. tuberculosis*, was revealed. Psoromic acid showed inhibitory impact on all the evaluated strains, with MIC values lower than isoniazid (INH), the standard drug. Psoromic acid showed remarkable suppression against UGM and significant inhibitory activity against TBNAT compared to INH. In silico analysis agreed with in vitro assays. Molecular interactions of PA with the UGM and TBNAT active sites were uncovered using molecular docking and structure-activity relationship studies.

Researchers investigated the effect of *Cladonia uncialis* acetone extract (CUE) and UA enantiomers on 10 skin infection-causing clinical microbial strains. At the same concentrations, (–)-UA alone was less active than CUE containing (–)-UA and squamatic acid. *Cladonia uncialis* acetone extract exhibited an activity that was similar to that of (+)-UA observed for *S. epidermidis* and *E. faecium *but did not display any activity against fungal strains. In addition, CUE showed low cytotoxicity against HaCaT (immortal human keratinocyte line) keratinocytes, compared to UA enantiomers, which is essential for its therapeutic use.^22^

The extracts of 12 lichen species were tested for their antibacterial potential by broth microdilution assay against 5 bacterial strains. None of the extracts were effective against gram (−) *E. coli*. On the other hand, *Cladonia borealis*, *Cladina confusa*, *Stereocaulom ramulosum,* and *Canoparmelia cryptochlorophaea *had 7.8 µg/mL MIC values against clinical isolates of *S. aureus* (resistant to clindamycin, erythromycin, and penicillin G) and *E. faecium* (resistant to vancomycin).^23^

### Antiviral Activity

Lai et al^24^ explored activity of sekikaic acid (SA) isolated from *Ramalina farinacea* on the respiratory syncytial virus. They found that sekikaic acid strongly (IC50: 5.69 µg/mL) inhibited a recombinant rg respiratory syncytial virus strainand respiratory syncytial virus A2 strain (IC50: 7.73 µg/mL). In addition, they investigated viability of HEp2 and Vero cells treated with sekikaic acid. The time of addition assay uncovered a clear interference of sekikaic acid with viral replication at a viral post-entry step. Sekikaic acid was more effective than the control ribavirin (over 1.3-fold), at 4 hours post-infection addition.

Hassan et al^25^ investigated HSV-1 DNA polymerase, herpes simplex virus type 1 (HSV-1), and type 2 HSV-2 inhibitory properties of PA. Cytotoxicity of the compound was tested in Vero cells. The authors also tested acyclovir (ACV) alone and in combination with PA. The results showed that PA significantly inhibited HSV-1 (IC50: 1.9 µM) and HSV-2 replication (EC50: 2.7 µM). The combination of PA and ACV further enhanced inhibition and selectivity in both viruses. Herpes simplex virus type 1 DNA polymerase activity of PA (IC50: 0.7 µM) was slightly better than ACV triphosphate (IC50: 0.9 µM) and aphidicolin (IC50: 0.8 µM). Researchers concluded that the inhibitory action of PA against HSV-1 DNA polymerase is involved in its inhibitory action on HSV-1.

### Enzyme Inhibition

Cheng et al^26^ reported that anziaic acid isolated from *Hypotrachyna *sp. was able to inhibit bacterial topoisomerase I. In *in vitro* assays, anziaic acid exhibited antibacterial activity against *Bacillus subtilis* and *E. coli*. The authors also found anziaic acid to function as an inhibitor of human topoisomerase II but had little effect on human topoisomerase I.

Luo et al^27^ isolated biruloquinone from *Cladonia macilenta* and it was shown to inhibit eel acetylcholinesterase with an IC50 value of 83.1 µM. According to the authors, biruloquinone was a mixed-II inhibitor on acetylcholinesterase and it improved the viability of the H_2_O_2_- and β-amyloid-injured PC12 cells at 1-25 µg/mL.

Bessadottir et al^28^ isolated (+)-protolichesterinic acid((+)-PA), a known anti-proliferative agent, from *Cetraria islandica* and tested whether the anti-proliferative activity of (+)-PA is associated with effects on fatty acid synthase (FASN), human epidermal growth factor receptor 2 (HER2), and major signaling pathways in SK-BR-3 and T-47D breast cancer cell lines. They revealed that treatment with (+)-PA increased FASN in SK-BR-3 cells, implying a compensatory response to the inhibition of this enzyme. Human epidermal growth factor receptor 2 expression was decreased suggesting secondary downregulation. ERK1/2 and AKT signaling pathways were inhibited, probably due to reduced levels of HER2.

Researchers evaluated the inhibitory potential of *Parmotrema tinctorum* ethyl acetate extract against α-glucosidase, aldose reductase (AR), and α-amylase. The extract was also evaluated in 2,2-diphenylpicrylhydrazyl (DPPH), 2,2'-azino-bis(3-ethylbenzothiazoline-6-sulfonic acid) (ABTS), superoxide, and hydroxyl radical-scavenging assays. It exhibited α-glucosidase, α-amylase, and AR inhibition, scavenged several free radicals, and thereby could reduce oxidative stress. Adding its protein glycation inhibition activity suggests that it can reduce postprandial hyperglycemia, diabetic retinopathy, and its associated complications.^29^

Cornejo et al^30^ isolated parietin from *Ramalina terebrata* and found that it could moderately inhibit the aggregation of tau protein, which participates in Alzheimer’s disease. The inhibition was detected between the concentration of 10 and 100 µM.

Authors investigated inhibition effect of 5 lichen acids (diffractaic, evernic, lobaric, lecanoric, and vulpinic acid) on mitochondrial hioredoxin reductase purified from rat lung in vitro and found that lichen acids’ (particularly lecanoric and vulpinic acid) IC50 values were significantly lower than IC50 values of cisplatin and doxorubicin.^31^

Cakmak and Gulcin^32^ reported that UA had significant antioxidant activity in various tests and was a good inhibitor of eel acetylcholinesterase and butyrylcholinesterase.

Researchers found that UA was able to inhibit purified human paraoxonase, glutathione reductase, and glutathione S-transferase enzymes in a favorable fashion. The authors concluded that UA could be useful in drug development studies.^33^

Three new depsidones, parmosidones F and G and 8'-O-methylsalazinic acid, isolated from the whole thalli of *Parmotrema dilatatum* were tested for their α-glucosidase inhibitory activity in vitro. Benzylated depsidones parmosidone F and parmosidone G inhibited α-glucosidase, a target for type 2 diabetes treatment, with IC50 values of 2.2 and 4.3 μM, respectively. The positive control, acarbose had an IC50 value of 449 μM.^34^

Eight molecules isolated from *Stereocaulon evolutum* were evaluated for their inhibition of protein tyrosine phosphatase 1B, which is considered as a new drug target in type 2 diabetes and other diseases. Lobaric and norlobaric acids and anhydrosakisacaulon A exerted effective inhibition with IC50 values of 12.9, 15.1, and 16.1 µM, respectively, which are comparable to the positive control ursolic acid (IC50: 14.4 µM).^35^

Ethanolic extract of *Himantormia lugubri *and 4 isolated metabolites (UA, barbatolic acid, 5,7-dihydroxy-6-methylphthalide, and atranol) were evaluated for their in vitro enzyme inhibitory effects on acetylcholinesterase, butyrylcholinesterase, and tyrosinase. Authors found that ethanolic extract inhibited acetylcholinesterase (IC50: 12.38 *± *0.09 µg/mL), butyrylcholinesterase (IC50: 31.54 *± *0.20 µg/mL), and tyrosinase (IC50: 22.32 *± *0.21 µg/mL). Atranol was the best inhibitor of tyrosinase (IC: 50 7.25 µg/mL), while UA was the best inhibitor of acetylcholinesterase (IC: 50 2.21 µg/mL) and butyrylcholinesterase (IC: 50 4.36 µg/mL).^36^

### Anti-Carcinogenic/Anti-Tumoral Activity

Retigeric acid B isolated from *Lobaria kurokawae* has been shown to inhibit cell growth and induce apoptosis in androgen-independent prostate cancer cells. Using PC3 and DU145 cells as models, investigators revealed that the mechanism underlying this action was the inhibition of phosphorylation levels of IκBα and p65 subunit of nuclear factor (NF)-κB in a time- and dosage-dependent manner. The authors also analyzed antitumor activity of retigeric acid B in C57BL/6 mice carrying RM-1 homografts. Retigeric acid B inhibited tumor growth and triggered apoptosis mainly by suppressing NF-κB activity in tumor tissues.^37^

Researchers investigated the mechanisms of cytotoxicity of parietin, atranorin, UA, and gyrophoric acid on A2780 and HT-29 cancer cell lines. According to the results, UA and atranorin were able to initiate an extensive loss in the mitochondrial membrane potential, along with caspase-3 activation (only in HT-29 cells) and phosphatidylserine externalization in both tested cell lines. The authors concluded that UA and atranorin are activators of programmed cell death in A2780 and HT-29, possibly via the mitochondrial pathway.^38^

Vicanicin isolated from *Psoroma* *dimorphum* and PA isolated from *Rhizoplaca* *melanophthalma* were investigated for antigrowth and pro-apoptotic activity in androgen-sensitive (LNCaP) and androgen-insensitive (DU-145) human prostate cancer cells. Both compounds showed a dose–response relationship in the range of 6.25-50 µM concentrations in DU-145 and LNCaP cells, activating an apoptotic process.^39^

Xu et al^40^ found that hexane, ethyl acetate, and water extracts from *Umbilicaria esculenta*, an edible lichen, exhibited telomerase inhibitory activities. Especially, the water extract possessed both telomerase inhibitory activity and cancer cell proliferation inhibitory activity, with an IC50 value of 19.54 μg/mL. Authors noted that the water extract of *U. esculenta* can possibly be applied as a novel anticancer agent.

In another study, researchers isolated physciosporin (PHY) from *Pseudocyphellaria coriacea.* Physciosporin demonstrated substantial inhibition on human lung cancer cells in migration and invasion assays. Physciosporin treatment decreased N-cadherin protein and mRNA levels with simultaneous reductions in epithelial-mesenchymal transition markers such as snail and twist. Physciosporin also suppressed KITENIN (KAI1 C-terminal interacting tetraspanin)-mediated AP-1 activity in both the absence and presence of epidermal growth factor stimulation. Physciosporin increased the expression of metastasis suppressor gene, KAI1 and significantly decreased the metastasis enhancer gene, KITENIN. Especially, the activity of 3'-untranslated region of KITENIN was declined by PHY. Also, PHY diminished Cdc42 and Rac1 activities.^41^

The authors evaluated methanolic *Cetraria islandica* and *Vulpicida canadensis* extracts in terms of cytotoxic activity against human hepatocellular carcinoma (HepG2) and breast adenocarcinoma (MCF-7) cells. Neuroprotective effect was investigated with respect to the antioxidant properties of the extracts. Oxygen radical absorbance capacity (ORAC) and DPPH assays were performed, and intracellular reactive oxygen species (ROS) production, caspase-3 activity, malondialdehyde, and glutathione levels were assessed in astrocytes in a hydrogen peroxide-induced oxidative stress model. Results indicated that both extracts exerted protective activity in astrocytes and cytotoxic activity in cancer cells. The authors concluded that both extracts merited further investigation.^42^

Antitumor potential of olivetoric, physodic, and PAs was investigated on human glioblastoma multiforme (U87MG-GBM) cell lines and primary rat cerebral cortex (PRCC) cells. Investigators found that olivetoric acid was a promising agent with high toxicity against cancer cells and low toxicity against healthy cells. They also reported that all metabolites presented favorable antioxidant profiles (i.e., higher total antioxidant capacity in healthy cells and higher total oxidative status in cancer cells).^43^

Brisdelli et al^44^ investigated the influence of PA on anti-proliferative activity of doxorubicin in HeLa, SH-SY5Y, and K562 cells and found that PA synergically increased the in vitro activity of doxorubicin against HeLa cancer cell line but not against SH-SY5Y and K562 cells. Protolichesterinic acid-promoted increase in cytotoxicity in HeLa cells was caused by a pro-apoptotic effect and was associated with caspase-3, 8, and 9 activation.

Three doses (50, 100, and 200 mg/kg body weight) of diffractaic acid isolated from *Usnea longissima* were found to have anti-tumoral activity against Ehrlich ascites carcinoma cells inoculated to mice. The best activity was observed with 200 mg/kg dose. Diffractaic acid did not have significant effect on hematological parameters.^45^

In another study, the methanol extract from *Cladonia pocillum* was able to inhibit cell proliferation while inducing programmed cell death in MCF-7 human breast cancer cells. The extract was found to prevent the oxidative damage to some extent. Also, the methanol extract of *C. pocillum* had antimicrobial activity, although not more than that of the chloroform extract.^46^

Researchers investigated the cytotoxicity of atranorin, gyrophoric acid, and physodic acid on A375 melanoma cancer cells. The depsidone physodic acid showed a dose–response relationship in the range of 6.25-50 μM concentrations in A375 cells, activating an apoptotic process that probably involves the reduction of Hsp70 expression.^47^

Researchers evaluated the anticancer capacities of ramalin, a secondary metabolite from the Antarctic lichen *Ramalina terebrata*, in the human colorectal cancer cell line HCT116. Ramalin significantly suppressed proliferation and induced apoptosis in a dose-dependent manner. In addition, it inhibited the migration and invasion of colorectal cancer cells. Cellular data revealed that, at both transcription and translation level, ramalin caused a gradual increase in the expression of tumor protein p53 and its downstream gene cyclin-dependent kinase inhibitor 1A, while decreasing the expression of cyclin-dependent kinase 1 and cyclin B1 and induced cell cycle arrest in the gap 2/mitosis phase.^48^

Ethyl acetate extract of *Usnea longissima* prevented gastric and esophageal carcinogenesis induced in rats with N-methyl-N'-nitro-N-nitrosoguanidine (MNNG). The extract was not cytotoxic up to 2000 mg/kg dose. Its prominent anticarcinogenic activity at 50 and 100 mg/kg doses suggested that it was selective to the cancer tissue.^49^

In recent studies, researchers found lichen substances vulpinic^50^ and diffractaic^51^ acids to be promising inhibitors of thioredoxin reductase 1, an overexpressed enzyme in breast cancer cells. These substances also exerted potent antiproliferative and antimigration effects on MCF-7 and MDA-MB-453 cell lines.

Diffractaic, lobaric, and (+)-UAs were tested for proliferation change, oxidative status, and DNA damage potentials against human glioblastoma multiforme (U87MGGBM) and PRCC cells. Authors found that lobaric acid highly inhibited the proliferation of PRCC and U87MG cells. Diffractaic and (+)-UAs exerted cytotoxic activity on both cells. The latter 2 was speculated to have minimal damage to healthy cells while reducing the number of cancerous cells.^52^

Researchers isolated tsavoenones A, B, and C from *Parmotrema tsavoense*. They evaluated tsavoenones A for its cytotoxicity against K562 and HepG2 tumor cell lines using doxorubicin as a positive control. The results showed that tsavoenones A exerts a moderate cytotoxicity against human myelogenous leukemia K562 cell line with an IC50 value of 66 ± 1.7 µg/mL.^53^

Investigators showed that an unidentified polysaccharide extracted from *Umbilicaria esculenta* was able to inhibit the growth of A875 and A375 melanoma cells, without obvious toxicity to normal vascular endothelial cells. The generation of ROS in A875 cells was significantly elevated after 24 hours treatment with the polysaccharide. In addition, the expression of caspase-3 and -9 also increased as compared to the controlled group, which resulted in the apoptosis of A875 melanoma cells.^54^

Reddy et al^55^ isolated barbatic acid (BA) from *Usnea longissima* and assessed its cytotoxic activity against cancer cells. Barbatic acid manifested doxorubicin equivalent activity against A549 lung cancer cell line with IC50 of 1.78 µM and strong G0/G1 accumulation of cells. Poly ADP-ribose polymerase cleavage confirmed that it induced cytotoxic activity via apoptosis. The authors concluded that BA was a genuine cytotoxic agent against A549 cells and could be considered as a new drug candidate to be assessed on oncogene-specific aggressive lung cancers.

Hydroalcoholic *Candelariella vitellina* extract exhibited a mitochondrial P53-independent apoptotic effect with negative P53 expression and an elevated BAX/BCL2 ratio as well as upregulated CASP3 mRNA expression in human colon cancer cell line. The extract showed no or low cytotoxicity on normal human peripheral lymphocytes. Similarly, in vivo analysis conducted in female Swiss albino mice demonstrated the same pattern of anticancer potential but was dependent on the P53 expression. Additionally, *C. vitellina* induced antioxidative conditions in vitro and in vivo. The decreased invasion of tumor cells in vivo and increased apoptotic features in vitro and in vivo suggest the fair to robust apoptotic anticancer potential of *C. vitellina*.^56^

Researchers investigated apoptotic effects and proliferative properties of extracts of *Lobaria pulmonaria*, *Evernia divaricata, Cladonia fimbriata, Bryoria capillaris, Usnea* *florida, *and *Hypogymnia tubulosa* on prostate cancer cells. They reported that *Hypogymnia tubulosa* and* Evernia divaricata* showed significant apoptotic activity on prostate cancer cells even at low concentrations, which implies that it is worth pursuing the biologically active lead compounds of these extracts on prostate cancer in vitro.^57^

Ingelfinger et al.^58^ in their comprehensive study, investigated the pharmacological activities of 6 lichen extracts in relation to cancer and inflammation. They used 11 screening assays in vitro. Results revealed that *F. caperata* extracts induced Ca^2+^ signaling and *E. prunastri, F. caperata*, *P. furfuracea, *and *P. glauca* extracts reduced cell migration. *F. caperata* extract strongly diminished tumor cell survival. *E. prunastri, F. caperata*, and *P. furfuracea* extracts significantly reduced endothelial cell proliferation. The extracts did not inhibit inflammatory processes in endothelial cells. However, *E. prunastri, P. furfuracea, F. caperata, *and* P. glauca* extracts inhibited the pro-inflammatory activation of leukocytes*. *None of the lichen extracts showed any detrimental influence on the viability of healthy cells.

Researchers investigated the potential compounds responsible for the antileukemic activity of lichen *Teloschistes flavicans*. They isolated a new compound, flavicansone, and showed that flavicansone demonstrated the most significant inhibitory action against cell proliferation in HL-60 cells. It reduced the number of viable HL-60 cells to under 20% of control.^59^

Another research^60^ focused on investigating the oral cancer cell-killing properties of methanolic *Usnea barbata* extracts (MEUB) against oral cancer cells. Methanolic *Usnea barbata* extracts displayed preferential killing against several oral cancer cell lines (Ca9-22, OECM-1, CAL 27, HSC3, and SCC9) but demonstrated minimal effect on healthy cells (HGF-1). The authors reported that all MEUB-induced alterations in oral cancer cells were triggered by oxidative stress, which was validated by pretreatment with antioxidant N-acetylcysteine. They concluded that MEUB caused preferential killing of oral cancer cells and it was associated with oxidative stress, apoptosis, and DNA damage.

Shendge et al^61^ investigated the antioxidant and anticancer potential of a tropical lichen *Dirinaria consimilis* (DCME). In vitro antioxidant studies showed promising ROS and reactive nitrogen species scavenging potential of DCME. The in vitro antiproliferative study revealed that DCME was cytotoxic toward human breast cancer cell line MCF-7 (IC50: 98.58 ± 6.82 μg/mL) and non-toxic toward healthy human lung fibroblast cell line WI-38 (IC50: 685.85 ± 19.51 μg/mL). The flow cytometric analysis showed an increase in sub G1 population as well as early apoptotic populations dose dependently. The results from confocal microscopy showed the DNA fragmentation in MCF-7 upon DCME treatment. The Western blotting study revealed the induction of tumor suppressor protein, p53, which results in increased Bax/Bcl-2 ratio and activation of caspase-cascade pathways. The activation of caspase-3, -8, -9, and poly-ADP ribose polymerase (PARP) degradation led the authors to conclude that DCME induced apoptosis in MCF-7 through both intrinsic and extrinsic mechanisms.

Kumar et al^62^ investigated the anticancer effects of UA against human gastric adenocarcinoma AGS and gastric carcinoma SNU-1 cells. Usnic acid (10-25 μM) treatment to these cells caused a significant increase in mitochondrial membrane depolarization and apoptotic cells. Apoptosis induction was accompanied by an increase in the ratio of Bax : Bcl-2 expression and cleaved PARP. Authors reported that UA acted through ROS generation and DNA damage on human gastric cancer cells accompanied by upregulation of γH2A.X (Ser139) phosphorylation, DNA-PKcs, and p53.

Fang et al^63^ reported that usenamine A isolated from *Usnea longissima* was capable of reducing the cell viability of both basal-like breast cancer (MDA-MB-231 and Hs578T) and luminal breast cancer (MCF-7) cells dose dependently while having lower cytotoxicity against normal breast cells MCF-10A. Cell apoptosis was dose dependently induced by usenamine A, and usenamine A treatment resulted in increased expression levels of cleaved caspase-3 and decreased expression levels of Bcl-2. In addition, usenamine A treatment dose dependently decreased the expression of cyclin B1, Cdc2, and cyclin A.

In a recent study, Taş et al^64^ investigated the effects of PHY on energy metabolism and tumorigenicity of the human breast cancer (BC) cells MCF-7 (estrogen and progesterone positive BC) and MDA-MB-231 (triple negative BC). They found that PHY treatment significantly decreased the mRNA level of PGC1-α genes in both BC cells. In vivo tests revealed that PHY suppressed expression of potential target markers, including HIF-1α, β-catenin, cyclin D1, c-Myc, GLUT1, and PKM2 in mice inoculated with 4T1-iRFP cells. Authors concluded that PHY suppressed energy metabolism as well as tumorigenesis in BC. Especially, PHY stood as a promising therapeutic effect against hormone-insensitive BC (triple negative) by targeting energy metabolism.

Jóhannsson et al^65^ used T-47D (breast cancer) and AsPC-1 (pancreatic cancer) cells to investigate mechanisms underlying the anti-proliferative effect of PA. They assessed morphological changes, metabolomics, mitochondrial function, glucose/lactate levels, glutathione (GSH), ROS, and NADP/NADPH. Authors measured a reduction in oxidative phosphorylation and increased glycolysis leading to structural changes in mitochondria of both cell lines. The GSH-consuming mercapturic pathway processed and expelled PA from the cells. Redox balance and ROS levels were minimally affected. The authors concluded that metabolic function and mitochondrial structure were most markedly affected implying that the impact of PA might depend on mitochondrial fitness.

Researchers found that vulpinic acid (VA) significantly reduced the viability of melanoma cells without toxicity in human epidermal melanocytes through apoptosis and its anti-inflammatory activity. Vulpinic acid induced apoptosis in A-375 melanoma cells through G2/M arrest, considerably induced the expression of CASP1, CASP2, CASP3, CASP8, and CASP10. Results showed that VA induced both intrinsic and extrinsic pathways of apoptosis. Additionally, VA exerted a strong anti-inflammatory activity in melanoma cells.^66^

### Gastroprotective Activity

Gastroprotective and in vivo antioxidant activities of rhizonyl alcohol isolated from *Lobaria pulmonaria* were investigated in indomethacin-induced ulcer models in rats. This metabolite exhibited antiulcerogenic activity as well as strong effects on in vivo oxidative parameters. It increased GSH levels and SOD, GPX, and GST activity, decreased LPO levels and CAT, MPO, and GR activites.^67^

### Anti-Diabetic Activity

Previous studies^68-70^ focused on possible anti-diabetic effects of *Cetraria islandica *and *Pseudevernia furfuracea* extracts in streptozotocin-induced diabetes in rats. Although investigators recommended low-dose *C. islandica* for early intervention in type 1 diabetes mellitus, the extracts did not improve diabetes-related parameters in rats.

In a randomized clinical trial, Kershengolts et al^71^ investigated the effects of a *Cladonia* sp.-based biologically active food supplement (BAFS) on a population of 150 type 2 diabetes mellitus-diagnosed patients. The BAFS was mechanically-chemically-activated lichen thalli, a process that increased digestible carbohydrates to 8-fold of un-activated material and also mobilized other biologically active substances. One hundred patients received BAFS and 50 patients received placebo for 6 months. According to biochemical measurements taken in third and sixth months, there was a significant improvement in serum lipid indicators (particularly total cholesterol decreased from 7.2 to 6.2 mmol/L in the sixth month), fasting venous plasma glucose (decreased from 10.2 to 7.6 in the third and 7.8 mmol/L in the sixth month) and HbA1c percent (decreased from 9.8 to 7.6 in the third and 7.4 in the sixth month) in BAFS receiving group. An interesting result from this study was the decrease in body mass index from 31 to 30 in the third month and retained the same at the sixth month in BAFS group, while it decreased from 30 to 29 in third month in the placebo group and raised back to 30 in sixth month.

Researchers assessed the antihyperglycemic activity as well as the antihyperlipidemic capacity of *Evernia prunastri* in normal and streptozotocin-induced diabetic rats. The results revealed that both single and repeated oral doses of *E. prunastri* (60 mg/kg) significantly reduced blood glucose, triglycerides, and very-low-density lipoprotein levels in diabetic rats. Furthermore, repeated oral administration of *E. prunastri* for 7 days ameliorated the liver function by increasing its glycogen content and improving its histological architecture in treated diabetic rats. *E. prunastri *extract showed antioxidant activity and proved richness in phenolic acids and flavonoids.^72^

### Hepatoprotective Activity

Researchers investigated the possible effects of UA on liver metabolism in livers of male Wistar rats and found that UA stimulated oxygen consumption at low concentrations, diminished the cellular ATP levels, increased the cytosolic but diminished the mitochondrial NADH/NAD + ratio, strongly inhibited gluconeogenesis, stimulated glycolysis, fructolysis, glycogenolysis, and ammonia genesis, and inhibited ureogenesis.^73^

On the other hand, hepatoprotective effect of diffractaic acid isolated from *Usnea longissima* was investigated against hepatic damage induced by carbon tetrachloride, and according to the authors, 50 mg/kg daily dose of diffractaic acid could be considered to have hepatoprotective effect by ameliorating the studied biochemical parameters and tissue histological structures.^74^

In another study,^75^ researchers investigated hepatoprotective effect of hydroalcoholic *Cladonia* *rangiferina* extract in ethanol-induced liver damage in rats. The extract showed a significant restoration of altered biochemical parameters toward normal in both *in vitro* and *in vivo* conditions. It restored alkaline phosphatase (ALP), alanine transaminase (ALT), aspartate aminotransferase (AST), total protein content, γ-glutamyl transferase, glutathione S-transferase, glutathione, malondialdehyde, and glutathione reductase levels almost back to normal at 50 and 100 mg/kg bw dose. Authors concluded that *C. rangiferina* could be a good treatment for alcohol liver disease.

Kim et al^76^ investigated the hepatoprotective properties of ramalin (RM) isolated from *Ramalina terebrata*, against hepatic fibrosis in vitro and in vivo. Ramalin suppressed hepatic stellate cell (HSC) activation in vitro without any significant signs of adverse effects on the cells tested, and the accumulation of extracellular matrix (ECM) was dramatically reduced in the liver tissue. Authors concluded that RM could attenuate hepatic fibrosis progression in dimethyl nitrosamine (DMN)-induced rats, potentially via the Nrf2/ARE pathway.

### Immunomodulatory and Inflammation-Related Activities

The researchers examined in vitro ability of stereocalpin A isolated from *Ramalina terebrata* to suppress the tumor necrosis factor-alpha (TNF-α)-induced expression of adhesion molecules in vascular smooth muscle cells (VSMCs). Two hours of stereocalpin A treatment at nontoxic concentrations (0.1-10 μg/mL) inhibited cell adhesion and expression of adhesion molecules. Stereocalpin A reduced TNF-α-induced production of intracellular reactive oxygen species and phosphorylation of signal and response pathways. Stereocalpin A inhibited TNF-α-induced activation and translocation of nuclear factors. Authors speculated that stereocalpin A has the potential to exert a protective effect by modulating inflammation within the atherosclerotic lesion.^77^

Four polysaccharide fractions were isolated from *Umbilicaria esculenta. *They were mainly composed of glucose, galactose, and mannose with different molar ratio. In the in vitro immunomodulatory assay, all the polysaccharide fractions (20-500 μg/mL) were able to increase the NO production and phagocytic activity of RAW 264.7 cells in a dose-dependent manner.^78^ Four polysaccharide fractions were isolated from *Umbilicaria esculenta. *They were made up from different molar ratios of glucose, galactose, and mannose. Around 20-500 μg/mL doses of all 4 polysaccharides increased phagocytic activity and NO production in RAW 264.7 cells in a dose-dependent manner.^78^

Researchers isolated lobastin from *Stereocaulon alpinum *and examined its effect on the expression of vascular cell adhesion molecules (VCAM-1) induced by TNF-α in MOVAS-1 (the cultured mouse vascular smooth muscle cells). Lobastin was able to inhibit VCAM–TNF-α-induced expression of VCAM-1. Lobastin also inhibited TNF-α-stimulated production of intracellular ROS, abolished TNF-α-induced phosphorylation of p38 and ERK 1/2, but not JNK, and inhibited TNF-α-promoted NF-κB activation. In addition, lobastin suppressed TNF-α-induced IκB kinase activation, subsequent degradation of IκBα and nuclear translocation of p65 NF-κB. Authors interpreted that lobastin downregulated the TNF-α-mediated induction of VCAM-1 in vascular smooth muscle cells by inhibiting intracellular ROS generation of p38, ERK ½, and NF-κB signaling pathways and intracellular ROS generation. They concluded that lobastin could be an important regulator of inflammation in the atherosclerotic lesion and be a novel therapeutic drug for the treatment of atherosclerosis treatment.^79^

Yu et al^80^ isolated 16 metabolites from *Usnea longissima* and tested some of them for NO production inhibition in RAW 267.4 cells. Compounds useanol, 3,7-dihydroxy-1,9-dimethyldibenzofuran, and 2,5-dimethyl-1,3-benzenediol showed significant anti-inflammatory activity against NO production with IC50 values of 6.8, 3.9, and 4.8 μmol/L, respectively, compared with the positive controls curcumin (IC50 15.3 μmol/L) and indomethacin (IC50 42.9 μmol/L).

In a recent study,^81^ the inhibitory activity of ethyl acetate extract of *Bryoria sp.* on the proliferation of CD8(+) T cells and the mixed lymphocytes reaction was evaluated in vitro. Authors suggested that Bryoria sp. extract inhibited mixed lymphocytes reaction via the suppression of IL-2Rα expression in CD8(+) T cells, and the extract had the potential to be developed as an anti-immunosuppression agent for organ transplants.

Researchers synthesized 16 new (+)-UA-based triazole hybrids and evaluated their anti-inflammatory potential against the cytokine proteins TNF-α and IL-1β on human U937 cells. Intermediates 2a, 2b, 3a, and 3b and triazoles 4f, 4g, 4h, 5f, 5g, and 5h exhibited promising anti-inflammatory activity against the TNF-α with IC50 values between 1.40 and 5.70 μM, while IL-1β inhibitions were insignificant. Prednisolone’s IC50 value was 0.52 µM. Authors suggested that compounds 5f and 5h were possible candidates for novel anti-inflammatory drug development.^82^

In an extensive work, acetone extract of *Everniastrum vexans* was investigated for anti-migratory activity against human lung cancer cell A549. The extract induced a potent inhibitory activity. Atranorin was determined as the active compound and further analyzed in various tests. Authors reported that atranorin significantly inhibited tumorigenesis in vitro and in vivo, and atranorin may inhibit lung cancer cell motility and tumorigenesis by affecting AP-1, Wnt, and STAT signaling and suppressing RhoGTPase activity.^83^

The anti-inflammatory activities of lobaric acid and pseudodepsidones isolated from *Stereocaulon paschale* were investigated. Lobaric acid was found to inhibit the NF-κB activation and the secretion of pro-inflammatory cytokines (IL-1β and TNF-α) in lipopolysaccharide (LPS)-stimulated macrophages. Inhibition and docking simulation experiments provided evidence that lobaric acid binds to PPAR-γ between helix H3 and the beta sheet, similarly to partial PPAR-γ agonists. Authors concluded that lobaric acid reduced the expression of pro-inflammatory cytokines by blocking the NF-κB pathway via the activation of PPAR-γ. Activities of pseudodepsidones were not as strong as lobaric acid.^84^

Researchers used Maillard reaction-based fluorescence assay to evaluate the in vitro inhibitory effects of secondary lichen metabolites on the formation of pentosidine-like advanced glycation end products. Of the tested 37 natural and 5 synthetically modified compounds, 18 exhibited IC50 values in the range of 50-700 µM. Best inhibitions were demonstrated by variolaric, pannaric, and leprapinic acids (IC50 values 50, 60, and 80 µM, respectively).^85^

Longissiminone A was isolated from *Usnea longissima* and screened for its in vivo anti-inflammatory and anti-platelet aggregation activities. It showed moderate in vivo anti-inflammatory activity in paw edema compared to aspirin as well as moderately active against the aggregation induced by arachidonic acid at different doses.^86^ Authors isolated longissiminone A from *Usnea longissima* and evaluated its in vivo anti-platelet aggregation and anti-inflammatroy potential. It exhibited moderate in vivo anti-inflammatory effect in paw edema compared to aspirin and was moderately active against the aggregation induced by arachidonic acid at different doses.^86^

In a recent study,^87^ the effect of lobaric acid isolated from *Stereocaulon alpinum* and its mechanism on LPS-induced inflammatory responses in macrophages were investigated. Researchers revealed that lobaric acid decreased nitric oxide production and the expression of cyclooxygenase-2 and prostaglandin E2 in LPS-stimulated macrophages. Significant reduction in the production of TNF-α and interleukin (IL)-6 was also recorded, and the underlying mechanism was suggested to inhibit the activation of mitogen-activated protein kinases (MAPKs) and nuclear factor-kappa B (NF-κB). Additionally, lobaric acid inhibited NLRP3 inflammasome activation in LPS/ATP-stimulated cells and extended the production of IL-1β and IL-18, as well as caspase1 maturation. Authors’ interpretation was that LA could inhibit inflammation by downregulating NF-κB/MAPK pathways and NLRP3 inflammasome activation in activated macrophages.

Researchers tested the antiarthritic property of methanolic extract of *Parmotrema tinctorum* in Freund’s complete adjuvant (CFA)-induced arthritic rat model. They investigated effects of the extracts on paw edema, arthritic score, radiological analyzes, blood parameters, and tissue enzyme and marker analyses. Constant administration of extract diminished the complications associated with arthritis by inhibiting the edema formation and arthritic score considerably. The altered biochemical parameters were restored with an enhancement in free radical scavenging ability after treatment. The authors reported that levels of especially TNF-α, C-reactive protein, and rheumatoid factor (RF) along with ALP have reverted to near normal.^88^

Researchers isolated a polysaccharide, CSL-0.1, from *Usnea longissima* and evaluated its effects on intestinal immunity and antioxidant activity. According to the results, CSL-0.1 dose dependently increased the spleen and thymus indices and presented immunomodulation on reversing the Th1/Th2-related cytokine imbalance in cyclophosphamide (CP)-induced immunosuppressed mice. CSL-0.1 also enhanced the levels of secretory immunoglobulin A in CP-injected mice. Additionally, the antioxidant levels in the liver and intestine of the mice were increased from 20% to 50% after intragastric injection by CSL-0.1.^89^

Kim et al^90^ investigated the anti-inflammatory effect of methanolic *Amandinea* sp. extract. The extract showed minimal cytotoxicity and dose dependently reduced NO production in LPS-stimulated RAW 264.7 cells. Investigators discovered that the extract inhibited the production of NO, IL-6, TNF-α, iNOS, and COX-2 in LPS-stimulated Raw 264.7 cells. They found that *Amandinea *sp. extract contained components that inhibit inflammation reactions through NF-κB signaling. The extract reduced the amount of ROS in LPS-induced zebrafish larvae and inhibited the mRNA expression of inflammatory cytokines and mediators in a tail-cutting-induced model.

Authors evaluated the anti-inflammatory potential of *Heterodermia hypoleuca*-derived secondary metabolite atraric acid. The outcomes showed that atraric acid was able to adjust promoted proinflammatory cytokine, prostaglandin E2, nitric oxide, and cyclooxygenase-2 and stimulated nitric oxide synthase enzyme expression in LPS-induced RAW264.7 cells. In addition, it downregulated the expression of extracellular signal-regulated kinases (ERK), phosphorylated IκB, and nuclear factor kappa B (NFκB) signaling pathway and demonstrated anti-inflammatory effects in LPS-stimulated RAW264.7 cells. Atraric acid’s in vivo anti-inflammatory impact was assessed in the LPS-stimulated mouse endotoxin shock model. It again downregulated inflammatory cytokines and decreased vasodilation and bleeding in damaged organs.^91^

Researchers isolated stereocalpin A, stereocalpin B, and an unnamed dibenzofuran (CAS registry number 674786-23-3) from *Ramalina terebrata* and investigated their biological activities. They found that all isolated substances exhibited moderate antimicrobial activities against *E. coli*, with the IC50 values ranging from 18 to 30 µg/mL. Stereocalpin A exhibited cell growth inhibition against HCT116 cell lines, with the IC50 value of 20 *± *1.20 µM. Both stereocalpins also showed potent anti-inflammatory activities against LPS-induced RAW 264.7 macrophages with the IC50 values ranging from 5 to 7 µM.^92^

### Neuroprotective Activity

Researchers reported the neurotrophic activity of atranorin (ATR), perlatolic acid (PER), PHY, and (+)-UA in a Neuro2A neurite outgrowth-based preliminary screening. No cytotoxicity was observed except for UA. Perlatolic acid also exerted acetylcholinesterase inhibition and strong proneurogenic activity. Atranorin, PER, and PHY modulated the gene expression of brain-derived neurotrophic factor and nerve growth factor. Perlatolic acid also showed increased protein levels of acetyl H3 and H4 in Neuro2A cells. These lichen substances showed neuroactive properties in vitro (Neuro2A cells) and ex vivo (primary neural stem or progenitor cells), indicating their potential to treat central nervous system disorders.^93^

Sieteiglesias et al^94^ evaluated the in vitro neuroprotective activities of methanol extracts of 15 lichens and underlying molecular mechanisms. SH-SY5Y cells (human neuroblastoma cell line) were used to elucidate neuroprotective activity. Of the tested extracts, *Parmotrema perlatum* and *Hypotrachyna formosana* improved cell viability in Fenton reagent-treated SH-SY5Y cells. The results demonstrated that *Parmotrema perlatum* and, especially, *Hypotrachyna formosana* are promising multitargeted neuroprotective agents. This neuroprotection was mediated by the reduction of ROS and lipid peroxidation.

Cazarin et al^95^ investigated UA enantiomers regarding cognitive-enhancing and anti-neuroinflammatory effects. The interaction of UA and acetylcholinesterase (AChE) was assessed by molecular docking and its inhibitory capability on AChE was assessed in vitro. In vivo effects of UA enantiomers were investigated in mice intracerebroventricularly (i.c.v.) exposed to Aβ1−42 peptide (400 pmol/mice). Additionally, UA antioxidant capacity and neuroinflammatory biomarkers were measured in the cortex and hippocampus of mice. The results revealed that UA enantiomers evoked complex–receptor interaction with AChE-like galantamine in silico. Usnic acid improved the learning and memory of the animals and in parallel decreased the myeloperoxidase activity and the lipid hydroperoxides (LOOH) in the cortex and hippocampus and reduced the IL-1β levels in the hippocampus.

Studzińska-Sroka et al^96^ used A-172, T98G, and U-138 MG glioblastoma cell lines to evaluate *Hypogymnia physodes* (HP) acetone extract and its major component PHY in terms of anticancer and neuroprotective activity. *Hypogymnia physodes *and PHY were able to strongly inhibit cell proliferation and hyaluronidase activity of glioblastoma. *Hypogymnia physodes* reduced tyrosinase and COX-2 activity. However, the acetylcholinesterase and butyrylcholinesterase inhibitions exerted by HP and PHY were moderate. Researchers demonstrated that PHY crossed the blood–brain barrier. They concluded that PHY and *Hypoymnia physodes* should be considered as promising agents with anticancer, chemopreventive, and neuroprotective activities, especially concerning the central nervous system diseases.

### Lipid Metabolism

Zhu et al^97^ targeted to uncover the impacts of water and ethyl alcohol extracts of *Usnea diffracta* on the adjustment of lipid metabolism, investigate the possible mechanism, and compare the effects with those of simvastatin. Rats were fed a high-fat diet for 45 days to induce hyperlipidemia. In serum, the extracts significantly reduced levels of total cholesterol, triglyceride (TG), and low-density lipoprotein cholesterol (LDL-C) and significantly increased the contents of high-density lipoprotein cholesterol. They decelerated weight gain and reduced TG and LDL-C content in liver. Serum levels of ALT and AST were reduced by the extracts. Total bile acid content in serum and liver was also reduced. In addition, researchers performed histopathological examinations and protein expression studies in liver tissues. They found that extracts could relatively improve cell degeneration and significantly reduce protein expressions of sterol regulatory element-binding proteins-1c and liver X receptor α (LXR-α). Furthermore, aqueous extract could substantially increase hepatic lipase activity and promote apoprotein A5 (ApoA5) protein expression. The authors concluded that ethanol extract exhibited higher activity and needed further studies, while aqueous extract could regulate lipid metabolism through increased ApoA5 expression via inhibition of the LXR-α signal pathway.

### Bone Metabolism

Kim et al^98^ studied whether UA affected receptor activator of NF-κB ligand (RANKL)-mediated osteoclastogenesis and found that it significantly inhibited RANKL-mediated osteoclast formation and function by reducing the transcriptional and translational expression of nuclear factor of activated T cells, cytoplasmic 1 (NFATc1), a master regulator of osteoclastogenesis. In addition, it prevented lipopolysaccharides-induced bone erosion in mice.

## Conclusions

In this review, experimental papers from the last 11 years about pharmaceutical activities of lichens were covered. It is evident that early papers from this period focused on antioxidant and anti-genotoxic activities of lichens. The focus shifted to anti-cancer activities and later enzyme inhibitory activities received remarkable attention.

There are topics that deserve much more attention and nano technologic use of lichens is absolutely one of them. The reader is referred to Hamida et al^99^ for a comprehensive review of the subject. Although the present review contains much data about anti-cancer activities of lichens, it is not possible to cover this subject along with other activities. Solarova et al^100^ recently reviewed this subject. Finally, ethnobotanical/ethnopharmacological aspect of lichens was not covered in this review, but very recently Adenubi et al^101^ published an excellent review on ethnobotanical uses of lichens.

## Figures and Tables

**Table 1.** Summary of Pharmacological Activities of Lichen-Derived Extracts and Compounds

Lichen Species	Extracts or Compounds	Compound Class	Activity
*Amandinea* sp	Ex	—	9
*Bacidia stipata*	Atranorin	Depside	5
*Bryoria capillaris*	Alectorialic acid	Depside	2
*Bryoria capillaris*	Barbatolic acid	Depside	2
*Bryoria *sp	Ex	—	9
*Candelariella vitellina*	Ex	—	5
*Canoparmelia cryptochlorophaea*	Ex	—	2
*Cetraria aculeata*	Ex	—	1
*Cetraria islandica*	Ex	—	5, 7
*Cetraria islandica*	Protolichesterinic acid	Cycloaliphatic acid	4, 5
*Cetrelia olivetorum*	Ex	—	1
*Cladina confusa*	Ex	—	2
*Cladonia borealis*	Ex	—	2
*Cladonia chlorophaea*	Ex	—	1
*Cladonia foliacea *	Ex	—	1
*Cladonia macilenta*	Biruloquinone	Quinone	4
*Cladonia pocillum*	Ex	—	5
*Cladonia* *rangiferina*	Ex	—	8
*Cladonia uncialis*	Ex	—	2
*Corncularia aculeata*	Protolichesterinic acid	Cycloaliphatic acid	5
*Dirinaria consimilis*	Ex	—	5
*Evernia divaricata*	Ex	—	5
*Evernia mesomorpha*	Divaricatic acid	Depside	2
*Evernia prunastri*	Ex	—	5, 7
*Everniastrum vexans*	Atranorin	Depside	9
*Everniastrum vexans*	Ex	—	9
*Flavoparmelia caperata*	Ex	—	5
*Heterodermia hypoleuca*	Atraric acid	Monocyclic Aromatic	9
*Himantormia lugubri*	5,7-dihydroxy-6-methylphthalide	Monocyclic Aromatic	4
	Atranol	Monocyclic Aromatic	4
	Barbatolic acid	Depside	4
	Usnic acid	Dibenzofuran	4
*Hypogymnia lugubris*	Physodic acid	Depsidone	5
*Hypogymnia physodes*	Ex	—	10
*Hypogymnia physodes*	Physodic acid	Depsidone	10
*Hypogymnia tubulosa*	Ex	—	5
*Hypotrachyna formosana*	Ex	—	10
*Hypotrachyna *sp	Anziaic acid	Depside	4
*Letharia columbiana*	Ex	—	2
*Letharia vulpina*	Ex	—	1,2
*Lobaria kurokawae*	Retigeric acid B	Terpenoid	5
*Lobaria pulmonaria*	Rhizonyl alcohol	Monocyclic Aromatic	6
*Ochrolecia deceptionis*	Gyrophoric acid	Depside	5
*Parmotrema dilatatum*	Parmosidone F	Depsidone	4
*Parmotrema dilatatum*	Parmosidone G	Depsidone	4
*Parmotrema perlatum*	Ex	—	10
*Parmotrema tinctorum*	Ex	—	4, 9
*Parmotrema tsavoense*	Tsavoenone A	Depside	5
*Peltigera horizontalis*	Ex	—	1
*Peltigera praetextata *	Ex	—	1
*Platismatia glauca*	Ex	—	5
*Pseudevernia furfuracea*	Ex	—	5
*Pseudevernia furfuracea*	Olivetoric acid	Depside	1, 5
*Pseudevernia furfuracea*	Physodic acid	Depsidone	1, 5
*Pseudocyphellaria coriacea*	Physciosporin	Depsidone	5
*Psoroma* *dimorphum*	Vicanicin	Depsidone	5
*Ramalina farinacea*	Sekikaic acid	Depside	3
*Ramalina terebrata*	Parietin	Quinone	4
	Ramalin	Monocyclic aromatic	5, 8
	Stereocalpin A	Cyclic depsipeptid	9
	Stereocalpin B	Cyclic depsipeptid	9
*Rhizoplaca marginalis*	Ex	—	2
*Rhizoplaca* *melanophthalma*	Protolichesterinic acid	Cycloaliphatic acid	5
	Psoromic acid	Depsidone	5
*Stereocaulom ramulosum*	Ex	—	2
*Stereocaulon alpinum*	Lobaric acid	Depsidone	9
	Lobastin	Pseudodepsidone	9
*Stereocaulon evolutum*	Anhydrosakisacaulon A	Diphenylether	4
	Lobaric acid	Depsidone	4
	Norlobaric acid	Depsidone	4
*Stereocaulon paschale*	Lobaric acid	Depsidone	9
*Teloschistes flavicans*	Flavicansone	Depsidone	5
*Umbilicaria esculenta*	Ex	—	5
*Umbilicaria esculenta*	Unidentified compounds	Polysaccharide s	5, 9
*Usnea albopunctata*	Protocetraric acid	Depsidone	2
*Usnea barbata*	Ex	—	5
*Usnea diffracta*	Ex	—	11
*Usnea hirta*	Ex	—	2
*Usnea longissima*	2,5-dimethyl-1,3-benzenediol	Moncyclic Aromatic	9
	3,7-dihydroxy-1,9-dimethyldibenzofuran	Dibenzofuran	9
	Barbatic acid	Depside	5
	CSL-0.1	Polysaccharides	9
	Diffractaic acid	Depside	5, 8
	Ex	—	5
	Longissiminone A	Monocyclic Aromatic	9
	Useanol	—	9
	Usenamine A	Dibenzofuran	5
*Usnea orientalis*	Ex	—	2
*Usnea steineri*	Usnic acid	Dibenzofuran	2
*Vulpicida canadensis*	Ex	—	2, 5
*Vulpicida pinastri*	Ex	—	1
*Xanthoria parietina*	Parietin	Quinone	2
*Z*	Atranorin	Depside	5, 10
Z	Diffractaic acid	Depside	1, 4, 5
*Z*	Evernic acid	Depside	2, 4
*Z*	Gyrophoric acid	Depside	5
Z	Lecanoric acid	Depside	1, 4
*Z*	Leprapinic acid	Pulvinic acid drv.	9
Z	Lobaric acid	Depsidone	1, 4, 5
*Z*	Pannaric acid	Dibenzofuran	9
*Z*	Parietin	Quinone	5
*Z*	Perlatolic acid	Depside	10
*Z*	Physciosporin	Depsidone	5
*Z*	Physodic acid	Depsidone	10
*Z*	Psoromic acid	Depsidone	2, 3
*Z*	Usnic acid	Dibenzofuran	4, 5, 8,10, 12
*Z*	Variolaric acid	Depsidone	9
*Z*	Vulpic acid	Pulvinic acid drv.	2
Z	Vulpinic acid	Pulvinic acid drv.	1, 4, 5

Activity numbers 1: anti-mutagenic and antioxidant activity, 2: antimicrobial activity; 3: antiviral activity, 4: enzyme inhibition activity, 5: anti-carcinogenic/anti-tumoral activity; 6: gastroprotective activity, 7: antidiabetic activity, 8: hepatoprotective activity, 9: immunomodulatory and inflammation-related activity, 10: neuroprotective activity, 11: lipid metabolism, 12: bone metabolism.

Drv, derivative; Ex, extract; Z, origin not indicated.

## References

[1] Yang M Devkota S Wang L Scheidegger C . Ethnolichenology—The Use of Lichens in the Himalayas and Southwestern Parts of China. Diversity. 2021;13(7):330. (10.3390/d13070330)

[2] Huneck S Yoshimura I . Identification of Lichen Substances. 2012 Springer-Verlag:493.Berlin, Heidelberg, New York

[3] Anar M Orhan F Alpsoy L Gulluce M Aslan A Agar G . The antioxidant and antigenotoxic potential of methanol extract of cladonia foliacea (huds.) willd. Toxicol Ind Health. 2016;32(4):721 729. (10.1177/0748233713504805) 24193055

[4] Anar m Aslan A Alpsoy L Kizil HE Agar G . Antigenotoxic and the antioxidant capacity of total extract of two lichens. Fresenius Environ Bull. 2016;25(3):684 691.

[5] Nardemir G Yanmis D Alpsoy L Gulluce M Agar G Aslan A Genotoxic, antigenotoxic and antioxidant properties of methanol extracts obtained from Peltigera horizontalis and Peltigera praetextata. Toxicol Ind Health. 2015;31(7):602 613. (10.1177/0748233713480207) 23456815

[6] Ceker S Orhan F Sezen S et al. Anti-mutagenic and anti-oxidant potencies of cetraria Aculeata (*Schreb.*) Fr., Cladonia Chlorophaea (*Flörke ex Sommerf.*) Spreng. and Cetrelia olivetorum (*Nyl.*) *W.L. Culb.* & *C.F. Culb.*). Iran J Pharm Res. 2018;17(1):326 335. 29755563 PMC5937102

[7] Kizil HE Ceker S Capik O et al. The anti-genotoxic effect of some lichenic acids. J Natl Sci Found Sri Lanka. 2018;46(2):227 231. (10.4038/jnsfsr.v46i2.8423)

[8] Emsen B Turkez H Togar B Aslan A . Evaluation of antioxidant and cytotoxic effects of olivetoric and physodic acid in cultured human amnion fibroblasts. Hum Exp Toxicol. 2017;36(4):376 385. (10.1177/0960327116650012) 27206701

[9] polat Z Aydın E Türkez H Aslan A . In vitro risk assessment of usnic acid. Toxicol Ind Health. 2016;32(3):468 475. (10.1177/0748233713504811) 24193043

[10] Lauinger IL vivas L perozzo R et al. Potential of lichen secondary metabolites against Plasmodium liver stage parasites with FAS-II as the potential target. J Nat Prod. 2013;76(6):1064 1070. (10.1021/np400083k) 23806111 PMC4119598

[11] Koçer S Uruş S Çakır A et al. The synthesis, characterization, antimicrobial and antimutagenic activities of hydroxyphenylimino ligands and their metal complexes of usnic acid isolated from Usnea longissima*. dalton* . Dalton Trans. 2014;43(16):6148 6164. (10.1039/c3dt53624f) 24589530

[12] Güven E Avcioğlu H Aslan A Hayirli A . Usnik Asitin Broylerlerdeki Anticoccidial Etkinliği. ­Kafkas Univ Vet Fak Derg. 2016;22(4):551 556. (10.9775/kvfd.2016.15008)

[13] Shrestha G Raphael J Leavitt SD St Clair LL . In vitro evaluation of the antibacterial activity of extracts from 34 species of North American lichens. Pharm Biol. 2014;52(10):1262 1266. (10.3109/13880209.2014.889175) 24863278

[14] Basile A Rigano D Loppi S et al. Antiproliferative, antibacterial and antifungal activity of the lichen Xanthoria parietina and its secondary metabolite parietin. Int J Mol Sci. 2015;16(4):7861 7875. (10.3390/ijms16047861) 25860944 PMC4425054

[15] Nishanth KS Sreerag RS Deepa I Mohandas C Nambisan B Protocetraric acid: an excellent broad spectrum compound from the lichen Usnea albopunctata against medically important microbes. Nat Prod Res. 2015;29(6):574 577. (10.1080/14786419.2014.953500) 25174415

[16] Pathak A Upreti DK Dikshit A . Antidermatophytic activity of the fruticose lichen Usnea orientalis. Medicines (Basel). 2016;3(3). (10.3390/medicines3030024) PMC5456250 28930134

[17] Tozatti MG Ferreira DS Flauzino LG et al. Activity of the lichen Usnea steineri and its major metabolites against gram-positive, multidrug-resistant bacteria. Nat Prod Commun. 2016;11(4):493 496. 27396202

[18] bellio P di pietro L Mancini A et al. Sos response in bacteria: inhibitory activity of lichen secondary metabolites against Escherichia coli reca protein. Phytomedicine. 2017;29:11 18. (10.1016/j.phymed.2017.04.001) 28515022

[19] Karagoz Y Karagoz K Dadasoglu F Ozturk-karagoz B . Bryoria capillaris (Ach.) Brodo & D. Hawksw. Extract fractions have potent antimicrobial activity in liquid - But not in solid media. Fresenius Environ Bull. 2018;27(6):4293 4297.

[20] Oh JM Kim YJ Gang HS Han J Ha HH Kim H . Antimicrobial activity of divaricatic acid isolated from the lichen Evernia mesomorpha against methicillin-resistant staphylococcus aureus. Molecules. 2018;23(12). (10.3390/molecules23123068) PMC6320781 30477128

[21] Hassan STS Šudomová M Berchová-Bímová K Gowrishankar S Rengasamy KRR . Antimycobacterial, enzyme inhibition, and molecular interaction studies of psoromic acid in mycobacterium tuberculosis: efficacy and safety investigations. J Clin Med. 2018;7(8). (10.3390/jcm7080226) PMC6111308 30127304

[22] Studzińska-Sroka E Hanna Tomczak Natalia Malińska et al.Cladonia uncialis as a valuable raw material of biosynthetic compounds against clinical strains of bacteria and fungi. Acta Biochim Pol. 2019;66(4):597 603. (10.18388/abp.2019_2891) 31837656

[23] Micheletti AC Honda NK Ravaglia LM Matayoshi T Spielmann AA . Antibacterial potencial of 12 lichen species. An Acad Bras Cienc. 2021;93(4). (10.1590/0001-3765202120191194) 34705932

[24] Lai D Odimegwu DC Esimone C Grunwald T Proksch P . Phenolic compounds with in vitro activity against respiratory syncytial virus from the Nigerian lichen Ramalina farinacea. Planta Med. 2013;79(15):1440 1446. (10.1055/s-0033-1350711) 23970423

[25] Hassan STS Šudomová M Berchová-Bímová K Šmejkal K Echeverría J . psoromic acid, a lichen-derived molecule, inhibits the replication of hsv-1 and hsv-2, and inactivates hsv-1 dna polymerase: shedding light on antiherpetic properties. Molecules. 2019;24(16). (10.3390/molecules24162912) PMC6720901 31405197

[26] Cheng B cao S Vasquez V et al. identification of anziaic acid, a lichen depside from hypotrachyna sp., as a new topoisomerase poison inhibitor. PLoS One. 2013;8(4):e60770. (10.1371/journal.pone.0060770) PMC3620467 23593306

[27] Luo H Li C Kim JC et al. biruloquinone, an acetylcholinesterase inhibitor produced by lichen-forming fungus cladonia macilenta. J Microbiol Biotechnol. 2013;23(2):161 166. (10.4014/jmb.1207.07016) 23412057

[28] Bessadóttir M Skúladóttir EÁ Gowan S Eccles S Ögmundsdóttir S Ogmundsdóttir HM . Effects of anti-proliferative lichen metabolite, protolichesterinic acid on fatty acid synthase, cell signalling and drug response in breast cancer cells. Phytomedicine. 2014;21(12):1717 1724. (10.1016/j.phymed.2014.08.006) 25442282

[29] Raj PS Prathapan A Sebastian J et al.Parmotrema tinctorum exhibits antioxidant, antiglycation and inhibitory activities against aldose reductase and carbohydrate digestive enzymes: an in vitro study. Nat Prod Res. 2014;28(18):1480 1484. (10.1080/14786419.2014.909420) 24735436

[30] cornejo A salgado F Caballero J Vargas R Simirgiotis M Areche C . Secondary metabolites in ramalina terebrata detected by UHPLC/ESI/MS/MS and identification of parietin as Tau protein inhibitor. Int J Mol Sci. 2016;17(8). (10.3390/ijms17081303) PMC5000700 27548142

[31] Ozgencli I Budak H Ciftci M Anar M . Lichen acids may be used as a potential drug for cancer therapy; by inhibiting mitochondrial thioredoxin reductase purified from rat lung. Anti Cancer Agents Med Chem. 2018;18(11):1599 1605. (10.2174/1871520618666180525095520) 29793415

[32] Cetin Cakmak K Gülçin İ . Anticholinergic and antioxidant activities of usnic acid-an activity-structure insight. Toxicol Rep. 2019;6:1273 1280. (10.1016/j.toxrep.2019.11.003) 31832335 PMC6889762

[33] Ceylan H Demir Y Beydemir Ş . Inhibitory effects of usnic and carnosic acid on some metabolic enzymes: an in vitro study. Protein Pept Lett. 2019;26(5):364 370. (10.2174/0929866526666190301115122) 30827223

[34] Devi AP Duong TH Ferron S et al. Salazinic acid-derived depsidones and diphenylethers with α-glucosidase inhibitory activity from the Lichen parmotrema dilatatum. Planta Med. 2020;86(16):1216 1224. (10.1055/a-1203-0623) 32819010

[35] Vu TH Delalande O Lalli C et al. Inhibitory effects of secondary metabolites from the lichen stereocaulon evolutum on protein tyrosine phosphatase 1B. Planta Med. 2021;87(9):701 708. (10.1055/a-1334-4480) 33618379

[36] Areche C Parra JR Sepulveda B García-Beltrán O Simirgiotis MJ . UHPLC-MS metabolomic fingerprinting, antioxidant, and enzyme inhibition activities of himantormia lugubris from antarctica. Metabolites. 2022;12(6). (10.3390/metabo12060560) PMC9227586 35736493

[37] Liu YQ Hu XY Lu T et al. Retigeric acid b exhibits antitumor activity through suppression of nuclear factor-κb signaling in prostate cancer cells in vitro and in vivo. PLoS One. 2012;7(5):e38000. (10.1371/journal.pone.0038000) 22666431 PMC3362538

[38] Bačkorová M Jendželovský R Kello M Bačkor M Mikeš J Fedoročko P . Lichen secondary metabolites are responsible for induction of apoptosis in ht-29 and a2780 human cancer cell lines. Toxicol In Vitro. 2012;26(3):462 468. (10.1016/j.tiv.2012.01.017) 22285236

[39] Russo A Caggia S Piovano M Garbarino J Cardile V . Effect of vicanicin and protolichesterinic acid on human prostate cancer cells: role of Hsp70 protein. Chem Biol Interact. 2012;195(1):1 10. (10.1016/j.cbi.2011.10.005) 22063921

[40] Xu B Li C Sung C . Telomerase inhibitory effects of medicinal mushrooms and lichens, and their anticancer activity. Int J Med Mushrooms. 2014;16(1):17 28. (10.1615/intjmedmushr.v16.i1.20) 24940901

[41] Yang Y Park SY Nguyen TT et al. Lichen secondary metabolite, physciosporin, inhibits lung cancer cell motility. PLoS One. 2015;10(9):e0137889. (10.1371/journal.pone.0137889) PMC4570789 26371759

[42] Fernández-Moriano C Divakar PK Crespo A Gómez-Serranillos MP . Neuroprotective activity and cytotoxic potential of two Parmeliaceae lichens: identification of active compounds. Phytomedicine. 2015;22(9):847 855. (10.1016/j.phymed.2015.06.005) 26220632

[43] Emsen B Aslan A Togar B Turkez H . In vitro antitumor activities of the lichen compounds olivetoric, physodic and psoromic acid in rat ­neuron and glioblastoma cells. Pharm Biol. 2016;54(9):1748 1762. (10.3109/13880209.2015.1126620) 26704132

[44] Brisdelli F Perilli M Sellitri D et al. Protolichesterinic acid enhances doxorubicin-induced apoptosis in hela cells in vitro. Life Sci. 2016;158:89 97. (10.1016/j.lfs.2016.06.023) 27363900

[45] Karagoz ID Ozaslan M Guler I et al. In vivo antitumoral effect of diffractaic acid from lichen metabolites on Swiss Albino mice with Ehrlich Ascites Carcinoma: an experimental study. Int J Pharmacol. 2014;10(6):307 314. (10.3923/ijp.2014.307.314)

[46] Ersoz M Coskun ZM Acikgoz B Karalti I Cobanoglu G Cesal C . In vitro evaluation of cytotoxic, anti-proliferative, anti-oxidant, apoptotic, and anti-microbial activities of Cladonia pocillum. Cell Mol Biol (Noisy-Le-Grand). 2017;63(7):69 75. (10.14715/cmb/2017.63.7.12) 28838343

[47] Cardile V Graziano ACE Avola R Piovano M Russo A . Potential anticancer activity of lichen secondary metabolite physodic acid. Chem Biol Interact. 2017;263:36 45. (10.1016/j.cbi.2016.12.007) 28012710

[48] Suh SS Kim TK Kim JE et al. Anticancer activity of ramalin, a secondary metabolite from the antarctic lichen ramalina terebrata, against ­colorectal cancer cells. Molecules. 2017;22(8). (10.3390/molecules22081361) PMC6152360 28817102

[49] Mammadov R Suleyman B Altuner d et al. Effect of ethyl acetate extract of usnea longissima on esophagogastric adenocarcinoma in rats. Acta Cir Bras. 2019;3;34. (10.1590/s0102-865020190030000005) PMC6585887 30892391

[50] Kalın ŞN Altay A Budak H . Inhibition of thioredoxin reductase 1 by vulpinic acid suppresses the proliferation and migration of human breast carcinoma. Life Sci. 2022;310:121093. (10.1016/j.lfs.2022.121093) 36270425

[51] Kalın ŞN Altay A Budak H . Diffractaic acid, a novel TrxR1 inhibitor, induces cytotoxicity, ­apoptosis, and antimigration in human breast cancer cells. Chem Biol Interact. 2022;361:109984. (10.1016/j.cbi.2022.109984) 35569514

[52] Emsen B Aslan A Turkez H Joughi AT Kaya A . The anti-cancer efficacies of diffractaic, lobaric, and usnic acid: in vitro inhibition of glioma. J Canc Res Ther. 2018;14(5):941 951. (10.4103/0973-1482.177218) 30197329

[53] Duong TH Beniddir MA Genta-Jouve G et al. Tsavoenones A-C: unprecedented polyketides with a 1,7-dioxadispiro[4.0.4.4]tetradecane core from the lichen Parmotrema tsavoense. Org Biomol Chem. 2018;16(32):5913 5919. (10.1039/c8ob01280f) 30074046

[54] Sun Y Li J Zhang Y et al. the polysaccharide extracted from Umbilicaria esculenta inhibits proliferation of melanoma cells through ros-activated mitochondrial apoptosis pathway. Biol Pharm Bull. 2018;41(1):57 64. (10.1248/bpb.b17-00562) 29311483

[55] Reddy SD Siva B Kumar K et al. Comprehensive analysis of secondary metabolites in usnea longissima (lichenized ascomycetes, parmeliaceae) using uplc-esi-qtof-ms/ms and pro-apoptotic activity of barbatic acid. Molecules. 2019;24(12). (10.3390/molecules24122270) PMC6630668 31216770

[56] El-Garawani IM Elkhateeb WA Zaghlol GM et al. candelariella vitellina extract triggers in vitro and in vivo cell death through induction of apoptosis: a novel anticancer agent. Food Chem Toxicol. 2019;127:110 119. (10.1016/j.fct.2019.03.003) 30853555

[57] goncu B Sevgi E Kizilarslan Hancer C Gokay G Ozten N . Differential anti-proliferative and apoptotic effects of lichen species on human prostate carcinoma cells. PLoS One. 2020;15(9):e0238303. (10.1371/journal.pone.0238303) 32997661 PMC7527208

[58] Ingelfinger R Henke M Roser L et al. Unraveling the pharmacological potential of lichen extracts in the context of cancer and inflammation with a broad screening approach. Front Pharmacol. 2020;11:1322. (10.3389/fphar.2020.01322) PMC7509413 33013369

[59] Sanjaya A Avidlyandi A Adfa M Ninomiya M Koketsu M . A new depsidone from Teloschistes flavicans and the antileukemic activity. J Oleo Sci. 2020;69(12):1591 1595. (10.5650/jos.ess20209) 33177283

[60] Tang JY Wu KH Wang YY et al. Methanol extract of Usnea barbata induces cell killing, apoptosis, and dna damage against oral cancer cells through oxidative stress. Antioxidants (Basel). 2020;9(8). (10.3390/antiox9080694) PMC7465944 32756347

[61] Shendge AK Panja S Basu T Mandal N . A tropical lichen, dirinaria consimilis selectively induces apoptosis in mcf-7 cells through the regulation of p53 and caspase-cascade pathway. Anti Cancer Agents Med Chem. 2020;20(10):1173 1187. (10.2174/1871520620666200318095410) 32188391

[62] Kumar K Mishra JPN Singh RP . Usnic acid induces apoptosis in human gastric cancer cells through ros generation and dna damage and causes up-regulation of dna-pkcs and γ-h2a.x phosphorylation. Chem Biol Interact. 2020;315:108898. (10.1016/j.cbi.2019.108898) 31715134

[63] Fang B Li Z Qiu Y Cho N Yoo HM . Inhibition of UBA5 expression and induction of autophagy in breast cancer cells by usenamine A. Biomolecules. 2021;11(9). (10.3390/biom11091348) PMC8469757 34572561

[64] Taş İ Varlı M Son Y et al. Physciosporin suppresses mitochondrial respiration, aerobic glycolysis, and tumorigenesis in breast cancer. Phytomedicine. 2021;91:153674. (10.1016/j.phymed.2021.153674) 34333327

[65] Jóhannsson F Cherek P Xu M . The anti-proliferative lichen-compound protolichesterinic acid inhibits oxidative phosphorylation and is processed via the mercapturic pathway in cancer cells. Planta Med. 2022;88(11):891 898. (10.1055/a-1579-6454) 34521132 PMC9439851

[66] Yangın S Cansaran-Duman D Eskiler GG Aras S . The molecular mechanisms of vulpinic acid induced programmed cell death in melanoma. Mol Biol Rep. 2022;49(9):8273 8280. (10.1007/s11033-022-07619-3) 35960408

[67] Atalay F Odabasoglu F Halici M et al. gastroprotective and antioxidant effects of lobaria Pulmonaria and its metabolite rhizonyl alcohol on indomethacin-induced gastric ulcer. Chem Biodivers. 2015;12(11):1756 1767. (10.1002/cbdv.201400432) 26567953

[68] Deniz GY Geyikoğlu F Türkez H Bakır TÖ Çolak S Aslan A . The biochemical and histological effects of lichens in normal and diabetic rats. Toxicol Ind Health. 2016;32(4):601 613. (10.1177/0748233713506769) 24193057

[69] Çolak S Geyikoğlu F Bakır TÖ Türkez H Aslan A . Evaluating the toxic and beneficial effects of lichen extracts in normal and diabetic rats. Toxicol Ind Health. 2016;32(8):1495 1504. (10.1177/0748233714566873) 25647809

[70] Bakir TO Geyikoglu F Colak S Turkez H Aslan A Bakir M . The effects of cetraria islandica and pseudevernia furfuracea extracts in normal and diabetic rats. Toxicol Ind Health. 2015;31(12):1304 1317. (10.1177/0748233713475521) 23833245

[71] Kershengolts BM Sydykova LA Sharoyko VV Anshakova VV Stepanova AV Varfolomeeva NA . lichens' b-oligosaccharides in the correction of metabolic disorders in type 2 diabetes mellitus. Wiad Lek. 2015;68(4):480 482. 26887115

[72] Amssayef A Bouadid I Eddouks M . Oakmoss exhibits antihyperglycemic activity in streptozotocin-induced diabetic rats. Cardiovasc Hematol Disord Drug Targets. 2022. (10.2174/1871529X22666220316100022) 35297355

[73] Moreira CT Oliveira AL Comar JF Peralta RM Bracht A . Harmful effects of usnic acid on hepatic metabolism. Chem Biol Interact. 2013;203(2):502 511. (10.1016/j.cbi.2013.02.001) 23422721

[74] Karagoz ID Ozaslan M Kilic IH et al. hepatoprotective effect of diffractaic acid on carbon tetrachloride-induced liver damage in rats. Biotechnol Biotechnol Equip. 2015;29(5):1011 1016. (10.1080/13102818.2015.1056754)

[75] Shukla I Azmi L Gupta SS . Amelioration of anti-hepatotoxic effect by lichen rangiferinus against alcohol induced liver damage in rats. J Ayurveda Integr Med. 2019;10(3):171 177. (10.1016/j.jaim.2017.08.007) 29395895 PMC6822147

[76] Kim MK Kim MA Yim JH Lee DH Cho SK Yang SG . Ramalin, an antioxidant compound derived from Antarctic lichen, prevents progression of liver fibrosis induced by dimethylnitrosamine (dnm) in rats. Biochem Biophys Res Commun. 2018;504(1):25 33. (10.1016/j.bbrc.2018.08.103) 30172374

[77] Byeon HE Park BK Yim JH et al. Stereocalpin a inhibits the expression of adhesion molecules in activated vascular smooth muscle cells. Int Immunopharmacol. 2012;12(2):315 325. (10.1016/j.intimp.2011.11.020) 22210374

[78] Du YQ Liu Y Wang JH . Polysaccharides from Umbilicaria esculenta cultivated in Huangshan mountain and immunomodulatory activity. Int J Biol Macromol. 2015;72:1272 1276. (10.1016/j.ijbiomac.2014.09.057) 25316425

[79] Lee K Yim JH Lee HK Pyo S . Inhibition of vcam-1 expression on mouse vascular smooth muscle cells by lobastin via downregulation of p38, erk 1/2 and NF-κB signaling pathways. Arch Pharm Res. 2016;39(1):83 93. (10.1007/s12272-015-0687-3) 26597859

[80] Yu XL Yang XY Gao XL et al. Phenolic constituents from lichen Usnea longissima. Zhongguo Zhong Yao Za Zhi. 2016;41(10):1864 1869. (10.4268/cjcmm20161017) 28895334

[81] Hwang YH Lee SJ Kang KY Hur JS Yee ST . Immunosuppressive effects of Bryoria sp. (lichen-forming fungus) extracts via inhibition of CD8(+) T-cell proliferation and IL-2 production in CD4(+) T cells. J Microbiol Biotechnol. 2017;27(6):1189 1197. (10.4014/jmb.1701.01080) 28372035

[82] Vanga NR Kota A Sistla R Uppuluri M . Synthesis and anti-inflammatory activity of novel triazole hybrids of (+)-usnic acid, the major dibenzofuran metabolite of the lichen Usnea longissima. Mol Divers. 2017;21(2):273 282. (10.1007/s11030-016-9716-5) 28130662

[83] Zhou R Yang Y Park SY et al. The lichen secondary metabolite atranorin suppresses lung cancer cell motility and tumorigenesis. Sci Rep. 2017;7(1):8136. (10.1038/s41598-017-08225-1) PMC5557893 28811522

[84] Carpentier C Barbeau X Azelmat J et al. Lobaric acid and pseudodepsidones inhibit NF-κB signaling pathway by activation of ppar-γ. Bioorg Med Chem. 2018;26(22):5845 5851. (10.1016/j.bmc.2018.10.035) 30420328

[85] Schinkovitz A Le Pogam P Derbré S et al. Secondary metabolites from lichen as potent inhibitors of advanced glycation end products and vasodilative agents. Fitoterapia. 2018;131:182 188. (10.1016/j.fitote.2018.10.015) 30339926

[86] Azizuddin Imran S Choudhary MI . In vivo anti-inflammatory and anti-platelet aggregation activities of longissiminone A, isolated from Usnea longissima. Pak J Pharm Sci. 2017;30(4):1213 1217 29039316

[87] Lee HW Kim J Yim JH Lee HK Pyo S . Anti-inflammatory activity of lobaric acid via suppressing NF-κB/mapk pathways or nlrp3 inflammasome activation. Planta Med. 2019;85(4):302 311. (10.1055/a-0777-2420) 30452073

[88] Syed Zameer Ahmed K Ahmed SSZ Thangakumar A Krishnaveni R . Therapeutic effect of Parmotrema tinctorum against complete freund's adjuvant-induced arthritis in rats and identification of novel isophthalic ester derivative. Biomed Pharmacother. 2019;112:108646. (10.1016/j.biopha.2019.108646) 30970506

[89] Wang T Shen C Guo F et al. characterization of a polysaccharide from the medicinal lichen, Usnea longissima, and its immunostimulating effect in vivo. Int J Biol Macromol. 2021;181:672 682. (10.1016/j.ijbiomac.2021.03.183) 33798588

[90] Kim JE Min SK Hong JM et al. Anti-inflammatory effects of methanol extracts from the Antarctic lichen, amandinea sp. in lps-stimulated raw 264.7 macrophages and zebrafish. Fish Shellfish Immunol. 2020;107(A):301 308. (10.1016/j.fsi.2020.10.017) 33068759

[91] Mun SK Kang KY Jang HY et al. Atraric acid exhibits anti-inflammatory effect in lipopolysaccharide-stimulated raw264.7 cells and mouse models. Int J Mol Sci. 2020;21(19). (10.3390/ijms21197070) PMC7582958 32992840

[92] Lee S Jeong SY Nguyen DL et al. Stereocalpin B, a new cyclic depsipeptide from the Antarctic lichen Ramalina terebrata. Metabolites. 2022;12(2). (10.3390/metabo12020141) PMC8880677 35208215

[93] Reddy RG Veeraval L Maitra S et al. Lichen-derived compounds show potential for central nervous system therapeutics. Phytomedicine. 2016;23(12):1527 1534. (10.1016/j.phymed.2016.08.010) 27765373

[94] Sieteiglesias V González-Burgos E Bermejo-Bescós P Divakar PK Gómez-Serranillos MP . Lichens of parmelioid clade as promising multitarget neuroprotective agents. Chem Res Toxicol. 2019;32(6):1165 1177. (10.1021/acs.chemrestox.9b00010) 31125207

[95] Cazarin CA Dalmagro AP Gonçalves AE et al. Usnic acid enantiomers restore cognitive deficits and neurochemical alterations induced by aβ(1-42) in mice. Behav Brain Res. 2021;397:112945. (10.1016/j.bbr.2020.112945) 33022354

[96] Studzińska-Sroka E Majchrzak-Celińska A Zalewski P et al. Permeability of hypogymnia physodes extract component-physodic acid through the blood-brain barrier as an important argument for its anticancer and neuroprotective activity within the central nervous system. Cancers (Basel). 2021;13(7). (10.3390/cancers13071717) PMC8038629 33916370

[97] Zhu J Zhang X Chen X et al. Studies on the regulation of lipid metabolism and the mechanism of the aqueous and ethanol extracts of Usnea. Biomed Pharmacother. 2017;94:930 936. (10.1016/j.biopha.2017.08.012) 28810520

[98] Kim KJ Jeong MH Lee Y et al. Effect of usnic acid on osteoclastogenic activity. J Clin Med. 2018;7(10). 10.3390/jcm7100345 PMC6210653 30322046

[99] Hamida RS Ali MA Abdelmeguid NE Al-Zaban MI Baz L Bin-Meferij MM . Lichens-A potential source for nanoparticles fabrication: a review on nanoparticles biosynthesis and their prospective applications. J Fungi (Basel). 2021;7(4):291. (10.3390/jof7040291) PMC8069866 33921411

[100] Solárová Z Liskova A Samec M Kubatka P Büsselberg D Solár P . Anticancer potential of Lichens’ secondary metabolites. Biomolecules. 2020;10(1):87. (10.3390/biom10010087) PMC7022966 31948092

[101] Adenubi OT Famuyide IM McGaw LJ Eloff JN . Lichens: an update on their ethnopharmacological uses and potential as sources of drug leads. J Ethnopharmacol. 20222022;298:115657. (10.1016/j.jep.2022.115657) 36007717

